# Metagenomic Characterization and Volatile Compounds Determination in Rumen from Saanen Goat Kids Fed Olive Leaves

**DOI:** 10.3390/vetsci9090452

**Published:** 2022-08-24

**Authors:** Francesca Bennato, Camillo Martino, Marco Di Domenico, Andrea Ianni, Benli Chai, Lisa Di Marcantonio, Cesare Cammà, Giuseppe Martino

**Affiliations:** 1 Faculty of Bioscience and Technology for Food, Agriculture and Environment, University of Teramo, 64100 Teramo, Italy; 2 Department of Veterinary Medicine, University of Perugia, 06126 Perugia, Italy; 3 Istituto Zooprofilattico Sperimentale dell’Abruzzo e del Molise “G. Caporale”, Via Campo Boario, 64100 Teramo, Italy; 4 Swift Biosciences, Ann Arbor, MI 48103, USA

**Keywords:** rumen microbiota, goat, olive leaves, 16s rRNA, volatile profile

## Abstract

**Simple Summary:**

The aim of this study was to characterize the rumen microbiota of Saanen goat kids fed olive leaves through a high-throughput approach based on 16S rRNA gene sequencing; furthermore, the parallel characterization of rumen volatile profile by solid-phase microextraction coupled with gas chromatography-mass spectrometry has been performed. Twenty goat kids were randomly assigned to two groups. The first group received a basal diet, while in the second one the diet was supplemented with olive leaves. The results showed the dietary supplementation to be able to affect the microbial community in the rumen. Significant differences were specifically observed between the two groups at genera and even family levels characterized by a higher abundance of cellulolytic bacteria in the rumen of goat kids fed olive leaves. In addition, the analysis of volatile compounds at the rumen level has allowed us to highlight differences in relation to the diet and the presence, in the rumen of goat kids fed olive leaves, of compounds indicative of health status.

**Abstract:**

The accumulation and disposal of by-products deriving from the agro-food industry represents a problem both from an economic and environmental point of view. The use of these matrices in zootechnical nutrition could represent a feasible solution. The aim of the study was to examine the effect of a diet containing olive leaves (OL), a by-product of the olive industry, on the ruminal microbial community of Saanen goat kids and on volatile organic compounds (VOCs) produced during the digestion. Twenty goat kids were randomly divided into two groups of ten goat kids each. The control group (CTR) was fed with a standard diet, while the experimental group (OL+) received a custom-formulated diet containing 10 % OL on a dry matter (DM) basis. After 30 days of trial, genomic DNA was extracted from the rumen liquor and prepared for 16S rRNA-gene sequencing to characterize the rumen microbiota; furthermore, rumen VOCs were also characterized by solid-phase microextraction coupled with gas chromatography-mass spectrometry. The Shannon’s alpha index was not significantly different between the two groups, on the contrary, Bray-Curtis (*p* < 0.01) and Jaccard (*p* < 0.01) distances evidenced that feed affected microbial community. Eleven genera were influenced by OL supplementation, with a significant increase (*p* < 0.05) in *Paludibacter*, *Fibrobacter, Sphaerochaeta Christensenella, Rikenella, Oligosphaera, Candidatus Endomicrobium, Anaerovorax,* and *Atopobium* was observed, while the percentages of *Bacteroides* and *Selenomonas* were reduced (*p* < 0.05). Differences were also observed between the two groups at the family level (*p* < 0.004). Fibrobacteriaceae, Christensenellaceae, Coriobacteriaceae, Oligosphaeraceae, Candidatus Endomicrobium, and Planctomycetaceae were significantly higher (*p* < 0.05) in goat kids fed OL diet compared to CTR, while the levels of other identified families, Succinivibrionaceae and Bifidobacteriaceae, were opposite (*p* < 0.05). Finally, results showed that the main phyla in both groups were Bacteroidetes and Firmicutes; however, no significant differences in the relative abundance of any phyla were observed between the two groups. In addition to what has been reported, the analysis of VOCs at the rumen level showed the ability of the OL integration to induce an increase in hexanoic acid and a parallel decrease in decanal. Furthermore, only in OL+ samples there was the accumulation of α-terpineol to which a wide range of interesting biological properties is attributed. The presence of VOCs associated with health status suggests a favorable role of OL in preserving and improving animal welfare.

## 1. Introduction

The rumen is an important digestive organ for ruminants, containing different types of bacteria, archaea, fungi, and ciliated protozoa [1,2]. These microorganisms play a key role in animal health, performance, milk, meat quality, and composition since they convert fibrous plant material into proteins, volatile fatty acids (VFAs), and vitamins to be used by the host animal to meet the requirement for essential processes such as growth, thermoregulation, and immunity [3]. Rumen microbial community could be affected by the animal species, breed [4], age [5,6], parity [7], and stage of lactation [8]. However, diet is the major determinant of ruminal microbial composition and metabolism, therefore, changes in the feeding strategy could alter the production of VFAs and methane, and ultimately influence the quality of meat and milk production [9,10,11]. 

During rumen fermentation of the feed, volatile organic compounds (VOCs) including several alcohols, VFAs, aldehydes, ketones, sulfides, and thiols can be produced by microbiological activity [12]. The characterization of these compounds is useful to evaluate the rumen microbial fermentations whose changes can be affected by animal health status, feed additives, and dietary strategy [13].

Olives are a major crop in Mediterranean countries. European, 2020–2021, olive oil production reached 2.3 million tons. It is estimated that 1.5–3 annual tons of leaves per ha can be collected during the pruning process. In the last years, a lot of research has focused on the recovery of by-products derived from olive oil extraction as olive pomace and also from olive tree pruning as olive leaves (OL). Several studies evidenced promising prospects in the use of olive by-products, fresh or treated, in small ruminant nutrition with effects on dairy products quality [14,15,16,17]. Of particular interest is the fact that OL are considered a natural and cheap source of phenolic compounds including oleuropein, tyrosol, hydroxytyrosol, caffeic acid, gallic acid, syringic acid, p-coumaric acid, and luteolin [18]. The information about the ability of OL to influence microbial fermentation processes in the rumen is limited and relative to in vitro studies. Shakeri et al. [19] showed a reduction by 15–53% of methane production, an increase in propionate production, and a significant reduction in acetate to propionate ratio in the rumen when OL and the different parts of the fruit were added to the in vitro incubations compared with the two different substrates used as control (oaten chaff and commercial concentrate). Molina-Alcaide et al. [20] reported a lower acetate-to-propionate ratio in continuous culture fermenters fed a diet containing OL (3.74 ratio) in comparison to a diet with olive leaves supplemented with barley grain (3.74 vs. 4.82 ratio, respectively). The addition of different concentrations of OL extract in the in vitro fermentation of a 50:50 forage:concentrate diet was tested by using a rumen simulation technique which evidenced a significant increase of 4–9% in the production of VFAs and propionate (11–14%), and a decrease in butyrate release (−8%) and total protozoa count (−8%) [21]. These findings suggested a role of OL dietary integration in improving some parameters associated with the fermentation efficiency in the rumen, with potential interesting effects on animal production. 

Changes in the rumen microbial populations and the identification of functional pathways activated in response to different feeding regimes can be efficiently studied through high-throughput sequencing and bioinformatic tools. In particular, 16S rRNA gene sequencing allowed identifying and quantifying the taxonomic composition of the rumen microbial population; for that reason, this approach was useful to predict the association between metagenome and metabolic functions using annotations collected in databases of microbial functional genes [22]. 

To our knowledge, no studies concerning the effect of OL on goat’s ruminal bacteria have been previously performed. Therefore, the aim of the present study was to identify possible changes due to OL dietary intake in goat kids rumen microbiota, using a metagenomic approach, and to characterize the production and accumulation of VOCs derived from microbial activity.

## 2. Materials and Methods

This study was performed on a commercial farm and animals were handled following the national legislation on animal welfare (Council Directive 2008/119/EC) [23], and then slaughtered in compliance with the Council Regulation 1099/2009 of the European Union [24] on the protection of animals at the time of killing. For the scope of the study, animals did not undergo breeding practices other than those commonly adopted; for this reason, it was not considered necessary to provide further ethical declarations.

### 2.1. Animal Management and Sampling

All twenty Saanen goat kids, homogeneous for age (90 d of age), sex (male), and weight (14.50 ± 1.65 kg), were randomly allotted into two groups (10 animals per group): a control group (CTR) and an experimental group (OL+) whose diet was integrated with OL collected from about 50 olive trees (*Olea* europaea), more than 50 years of age, belonging to Leccino variety and grown in a limited area of Abruzzo, Italy (Castellalto, Teramo, Italy). The OL chemical composition is reported in a previous study [25]. The trial took place in April and lasted 30 days. The goat kids were housed for the entire trial period in two separate areas of free housing with bunks on straw, a drinking trough, and access to an identical feeding area. The diets were formulated to be isoenergetic; every day, each goat kid of both the group received alfalfa hay administered ad libitum with 1 kg/head/d of a concentrate (Table 1). The diet of the experimental group was mixed with OL (10 g/100 g on a dry matter (DM) basis of the whole diet). OL harvested during the winter pruning were dried in a ventilated oven at 60 °C until reaching a moisture of 10%, ground, and mixed with the concentrate.

Diet samples were analyzed for DM (method 934.01), crude protein (CP; method 988.05), ether extract (EE; method 920.39), crude fiber (CF; method 962.09), and ash (method 942.05) according to AOAC methods (AOAC, 2000) [26]; neutral detergent fiber (NDF), acid detergent fiber (ADF), and acid detergent lignin (ADL) were determined by the detergent procedures of Goering and Van Soest [27].

Within 60–90 min from slaughtering (120 d of age), as reported by Biscarini et al. [22], 500 mL of the whole rumen content (consisting of a mixture of liquid and solid fractions) from the dorsal, central, and ventral of each goat kids was immediately collected, pooled, and filtered through four layers of cheesecloth, and then collected in 50 mL tubes and stored at −20 °C until further analysis (about 1 month).

### 2.2. DNA Extraction and Illumina MiniSeq Sequencing

Total microbial DNA was extracted and purified from the rumen liquid (*n* = 10 per group) by the Maxwell 16 LEV simplyRNA Blood Kit (Promega, Madison, WI) following the manufacturer’s instructions without using the DNaseI. Briefly, 1 mL of rumen fluid was transferred to 2 mL tubes where 0.5 mL of phosphate-buffered saline (PBS) was added. The tubes were then quickly mixed by vortex and centrifuged for 10 min at 300 g to pellet the vegetal matter. A volume of 300 μL supernatant was transferred to the cartridges provided by the kit followed by the automatic extraction/purification workflow on the Maxwell 16 instrument (Promega, Madison, WI). Then, DNA was quantified by Qubit dsDNA HS assay (Thermo Fisher Scientific, Waltham, MA) and diluted to 2 ng/μL for library preparation by means of the Swift Amplicon 16S + ITS panel Kit (Swift Bioscience, Ann Arbor, MI, USA) that targets all the hypervariable regions (V1-V9) of the bacterial 16S rRNA gene. DNA libraries were sequenced on the MiniSeq Illumina platform (Illumina, San Diego, CA, USA) according to the manufacturer’s instructions. A 20-strain even mix of bacterial genomic DNA (ATCC MSA-1002) was used as sequencing and bioinformatic analysis control.

### 2.3. Evaluation of Volatile Compounds in Rumen

The characterization of volatile organic compounds (VOCs) in goat kids rumen samples, was performed according to previous research [12,28], by using a divinylbenzene-carboxen-polydimethylsiloxane solid phase microextraction (SPME) fiber. Each sample of rumen was weighed and placed in a test tube. After pressing, 10 mL of rumen fluid was immediately transferred into a glass vial and mixed with 5 mL of saturated NaCl solution (360 g/L) and 10 μL of internal standard solution (4-methyl-2-heptanone; 10 mg/kg in ethanol). The vials were sealed with a polytetrafluoroethylene-silicone septum (Supelco, Bellefonte, PA, USA) and stirred for 30 min at 39 °C to simulate the rumen environment. The VOCs were analyzed by a gas chromatograph (Clarus 580; Perkin Elmer, Waltham, MA, USA) coupled with a mass spectrometer (SQ8S; Perkin Elmer). The gas chromatograph was equipped with an Elite-5MS column (length × internal diameter: 30 × 0.25 mm; film thickness: 0.25 μm; Perkin Elmer). The fiber was thermally desorbed into the GC injector for 30 min in a splitless mode at 250 °C. The column oven initial temperature was 50 °C (held for 1min), followed by a first ramp of 3 °C/min to reach 200 °C (held for 1 min) and a second ramp of 15 °C/min to obtain 250 °C (held for 15 min). Helium was used as the carrier gas with a flow rate of 1 mL/min. Analytes were identified as previously described [29]. The data were expressed as relative abundance, as a percentage of each compound on the sum of the total VOCs detected.

### 2.4. Bioinformatic and Statistical Analysis

Data processing was performed using the Swift 16S SNAPP open-source pipeline (https://github.com/swiftbiosciences/16S-SNAPP, accessed on 6 October 2020) [30], which used the Ribosomal Database Project (RDP) Classifier (Wang et al., 2007) for classifying consensus sequences. Statistical analysis was performed on abundance tables generated by 16S SNAPP using R scripts with packages including vegan (https://github.com/vegandevs/vegan, accessed on 6 October 2020) [31], ggvegan (https://github.com/gavinsimpson/ggvegan/, accessed on 6 October 2020) [32], and ggplot2 (https://github.com/tidyverse/ggplot2/, accessed on 6 October 2020) [33]. Low-count genera (<0.1% of total reads) were filtered out and sample size rarefied to the smallest sample size of 22,659 reads.

Alpha diversity was used to define the variety and the abundance of species in the rumen. The indices used to calculate alpha diversity were Shannon and Chao1. Bray-Curtis and Jaccard indices were used to analyze similarities or differences among the groups (beta diversity) at the genus, family, and phylum level. The contribution of individual taxa to the overall Bray-Curtis dissimilarity index was computed by SIMPER (similarity percentage, vegan). 

Permutational multivariate analysis of variance (Adonis) was used for beta diversity on both Bray-Curtis and Jaccard distances to evaluate quantitative and qualitative dissimilarity between the two groups, respectively. Bray-Curtis dissimilarity index ranges are from 0 to 1; values close to 0 mean experimental groups share the exact same number of each type of species and values close to 1 share none of the same kind of species. Conversely, the Jaccard similarity index varies from 0 to 1 (File S1). The closer to 1, the more similar the two sets of data. Moreover, the Kruskal–Wallis non-parametric ANOVA test was used for differential abundances between the two groups. With specific regard to VOCs analysis, the separation of means between the two groups was assessed by Student’s *t*-test, and differences were considered significant for *p* values lower than 0.05 and 0.01. 

## 3. Results

### 3.1. Acquisition of Metagenomic Data and Analysis

We obtained a mean of 236,831 and 277,576 reads for the CTR and OL+ group, respectively. From 75–97% starting reads matched primers. Over 4.1 Mb non-chimeric reads, 2.2 Mb (80%) from OL integration feed, and 1.9 Mb (83%) from conventional feed were used in generating consensus sequences for analysis (Table 2). All the taxa of the ATCC MSA-1002 were correctly classified at the genus level.

### 3.2. Effects of Diet on Microbial Community Composition

**Figure 1:**
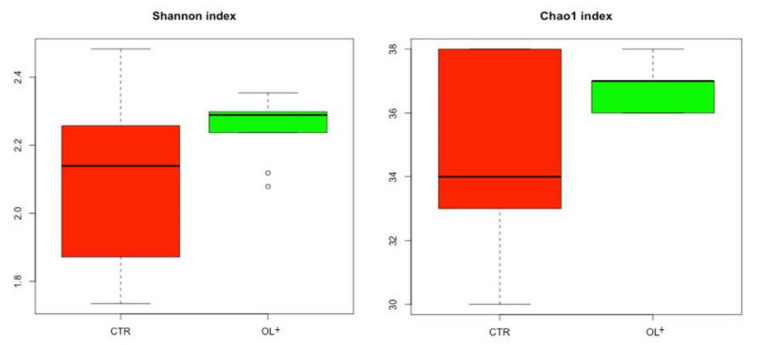
Alpha diversity between control group (CTR—red) and experimental group (OL+—green).

Alpha diversity (Shannon and Chao1) was calculated at the genus level between two feed groups OL+ and CTR (Figure 1). Although OL+ appears to be slightly higher overall than CTR, the differences were not statistically significant (*p* > 0.05).

Beta-diversity analysis showed OL+ and CTR are significantly different (Adonis tests of both Bray-Curtis and Jaccard indices, *p* ≤ 0.001). In this study, Bray-Curtis and Jaccard indices evidenced that OL integration in feed affects the rumen microbial community. The contribution of individual genera to the overall Bray-Curtis dissimilarity is reported in Table 3.

**Figure 2:**
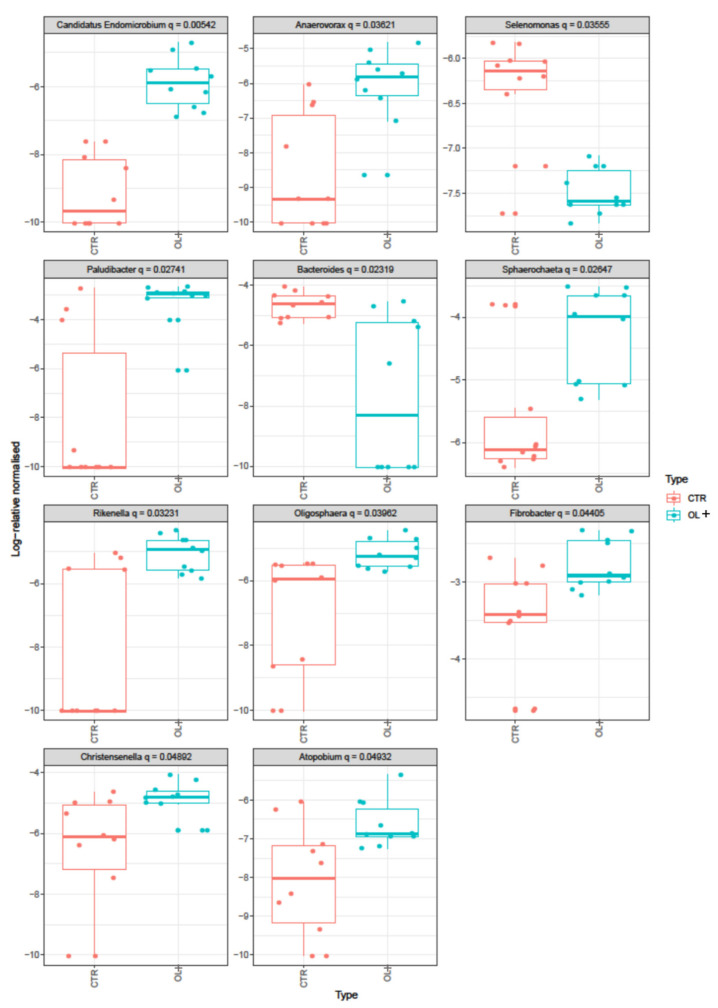
Boxplots showing the Bray-Curtis dissimilarities at genera level. The boxes in the plot represent the interquartile ranges, the horizontal lines give the position of the medians, the vertical bars indicate the range. The dots indicate outliers.

The analysis of the sequences identified a total of 157 genera in the two groups; however, the genera with a relative abundance greater than 1% were 16 for CTR and 14 for OL+ (Table S1). Percentage contribution to Bray-Curtis dissimilarity showed that 11 genera were influenced by OL integration (Figure 2). Among them, in OL+, a higher number of reads (*p* < 0.05) was observed in *Paludibacter* (259 counts vs 1061.5 counts in CTR and OL+, respectively), *Fibrobacter* (849.2 counts vs 1502.7 counts in CTR and OL+, respectively), *Sphaerochaeta* (142.3 counts vs 386.2 counts in CTR and OL+, respectively), *Christensenella* (76.1 counts vs 192.9 counts in CTR and OL+, respectively), *Rikenella* (44.4 counts vs 165.7 counts in CTR and OL+, respectively), *Oligosphaera* (49.7 counts vs 137 counts in CTR and OL+, respectively), *Candidatus Endomicrobium* (3.4 counts vs 77.6 counts in CTR and OL+, respectively), *Anaerovorax* (12.3 counts vs 71.3 counts in CTR and OL+, respectively), *Atopobium* (12.7 counts vs 40.6 counts in CTR and OL+, respectively). Conversely, OL integration reduced the percentage of *Bacteroides* (233.2 counts vs 67.3 counts in CTR and OL+, respectively) and *Selenomonas* (43 counts vs 11.6 counts in CTR and OL+, respectively).

Beta diversity was then calculated at family taxonomic rank with statistical significance slightly lower (*p* < 0.004) than genus-level tests. At this taxon level, the sequences could be assigned to thirty-two different families (Table 4).

**Figure 3:**
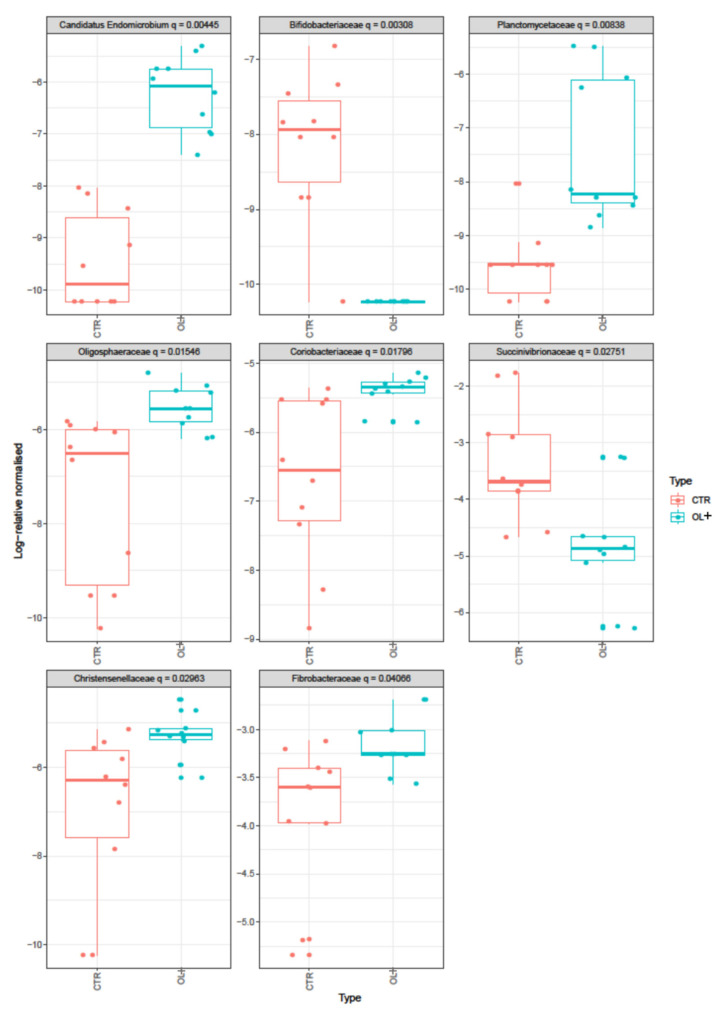
Boxplots showing the Bray-Curtis dissimilarities at family level. The boxes in the plot represent the interquartile ranges, the horizontal lines give the position of the medians, the vertical bars indicate the range. The dots indicate outliers.

Among them, 8 taxa changed significantly in the two groups (*p* < 0.01) (Figure 3). Fibrobacteriaceae (684 counts vs 1249.5 counts in CTR and OL+ respectively), Christensenellaceae (71.7 counts vs 165.2 counts in CTR and OL+, respectively), Coriobacteriaceae (67.3 counts vs 131.5 counts in CTR and OL+, respectively), Oligosphaeraceae (39 counts vs 121.3 counts in CTR and OL+, respectively), Candidatus Endomicrobium (2.6 counts vs 61.1 counts in CTR and OL+, respectively), and Planctomycetaceae (0.9 counts vs 36.1 counts in CTR and OL+, respectively) were observed at a higher level in OL+, while Succinivibrionaceae (1699.2 counts vs 347.1 counts in CTR and OL+, respectively) and Bifidobacteriaceae (7.3 counts vs 0 counts in CTR and OL+, respectively) were mostly represented in the CTR.

**Figure 4:**
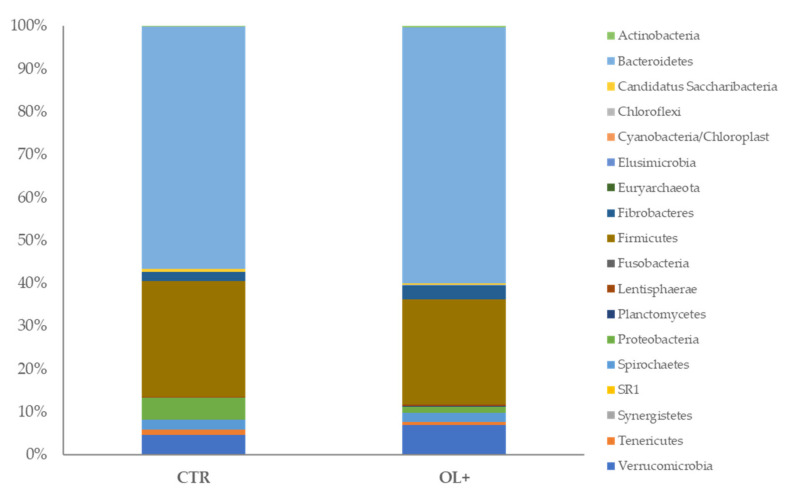
Phyla-level relative abundance. A color-coded bar plot showing the average bacterial phyla distribution across the control group (CTR) and experimental group (OL+).

Finally, at the phylum level, the overall differences between OL+ and CTR are not significant based on Adonis of both Bray-Curtis and Jaccard indices (*p* > 0.05). In both groups 18 phyla were identified; however, in CTR, 7 phyla had a relative abundance > 1%, on the contrary, in OL+ 6 phyla. In both groups, the most represented phyla were Bacteroidetes (56.40% vs 59.76% relative abundances in CTR and OL+, respectively), Firmicutes (26.88% vs 24.57% relative abundances in CTR and OL+, respectively), Fibrobacteres (2.09% vs 3.09% relative abundances in CTR and OL+, respectively), Proteobacteria (5.21% vs 1.45% relative abundances in CTR and OL+, respectively), Spirochaetes (2.25% vs 2.21% relative abundances in CTR and OL+, respectively), Verrumicrobia (4.71% vs 7.00% relative abundances in CTR and OL+, respectively), and Actinobacteria (0.24% vs 0.31% relative abundances in CTR and OL+, respectively). The relative abundance of Bacteroidetes was the highest in both groups (56.40% vs 59.76% in CTR and OL+, respectively) followed by Firmicutes (26.88% vs 24.57 % in CTR and OL+, respectively) which together represent 84.03% and 83.28% in OL+ and CTR, respectively (Figure 4). No differences were observed for the relative abundance of any phyla between the two groups (*p* > 0.05), as well as for the Firmicutes-to-Bacteroidetes ratio (0.4:1), which represents an indicator of lignocellulose breakdown capacity. 

### 3.3. Identification of Volatile Compounds

The analysis of VOCs in the rumen samples obtained from the goat kids made it possible to identify a total of 24 compounds belonging to different chemical families: acids, aldehydes, alcohols, ketones, esters, and hydrocarbons. As shown in Table 5, the OL introduction into the animal’s diet showed effectiveness in inducing some variations compared to the control group. Specifically, in OL+ samples, a higher concentration of hexanoic acid (7.07 ± 0.76% vs 9.35 ± 0.89% in CTR and OL+, respectively, *p* < 0.05) was found, while significantly lower values were found for the decanal (1.89 ± 0.17% vs 0.85 ± 0.10% in CTR and OL+, respectively, *p* < 0.01). Furthermore, the finding concerning the presence of α-terpineol (3.06 ± 0.26%) only in samples obtained following the OL dietary supplementation is peculiar.

## 4. Discussion

The rumen microbial community structure is affected by animal species, breed, age, parity, and lactation stage. However, diet is the major determinant of ruminal microbial composition, and changes in the feeding strategy can lead to rapid and dramatic changes in gut microorganisms [9,10,11]. Rumen microorganisms metabolize polysaccharides, proteins, and lipids in VFAs, microbial proteins, and vitamins that are used by animals [34]. To date, several metagenomic studies have been focused on the effect of different dietary protocols on the rumen microbiome. However, knowledge of microbial ecology is still limited and, in particular, the effects of dietary OL supplementation on the ruminal goat microbiome have not been reported in the literature. 

Independently from the diet, in the present study, the main phyla identified in the rumen were Bacteroidetes, followed by Firmicutes, whereas subdominant phyla were Proteobacteria, Verrumicrobia, Fibrobacteres, and Actinobacteria. This result is consistent with previous studies published by Cremonesi et al. [35], who reported that the rumen composition of Alpine dairy goats was dominated by Bacteroidetes with about 61.2% relative abundance, followed by Firmicutes, Proteobacteria, and Verrucomicrobia at 24.2%, 4.1%, and 3.3% relative abundances, respectively. Conversely, in free-ranging Moxotó breed goats, a lower relative abundance of Bacteroidetes and a high abundance of Firmicutes was observed in the rumen microbiome [36]. This aspect, on the one hand, may depend on the different genetics of the animals involved in these two experiments; on the other, it is certainly influenced by the diet of the animals, supporting the fact that the presence of different substrates at the rumen level is effective in changing the microbiota composition.

Members of phylum Bacteroidetes are the primary degraders of complex polysaccharides in the plant cell wall, and they have higher mean glycoside hydrolase (GH) enzymes and polysaccharide lipases (PLs) genes per genome as well as signal peptide-containing GH and PLs compared with the members of the phylum Firmicutes which have a lower ability for polysaccharide degradation and seem to possess more cellulose hydrolysis capacity. Moreover, they are known for their production of butyric acid or any other bacterial phyla in the gut intestinal tract [37,38].

The data obtained in the present study showed a low (0.4:1) Firmicutes/Bacteroidetes ratio in both groups. Similarly, in Vietnamese goat rumen, a dominance of Bacteroides compared to Firmicutes and a low Firmicutes/Bacteroidetes ratio correlated to a high diversity of cellulolytic GH enzymes originating from Bacteroidetes was observed [39]. The ratio between Firmicutes and Bacteroidetes is influenced by many factors such as breed, age, and diet [38]. During the life cycle of the goat, this ratio gradually decreases, changing from 2.1:1 at 80 days old to 1.7:1 at 100 days old and to 0.3:1 at 110 days old [40]. This decrease has been correlated to the adaptation of rumen bacteria to a plant diet. Recently, in goats fed a high-grain diet, it has been observed an increase in ruminal acidity and a very high Firmicutes/Bacteroidetes ratio (~3:1), which are thought to be unhealthy changes [41]. The data obtained in this study suggest that the percentage of OL integration did not interfere with the normal rumen ecosystem.

Regarding the taxonomy at the family level, in our samples, the highest percentage contributions to Bray-Curtis dissimilarity were Prevotellaceae, Subdivision5_genera_incertae_sedis, Succinivibrionaceae, and Ruminococcaceae. However, OL integration induced significant increases in different families: Fibrobacteraceae, Christensenellaceae, Coriobacteriaceae, Oligosphaeraceae, Candidatus Endomicrobium, and Planctomycetaceae; conversely, Succinovibrionaceae and Bifidobacteriaceae decreased. Among them, even if not significant, a higher abundance of Ruminococcaceae was detected in OL+ with respect to CTR. Ruminococcaceae are fibrolytic bacteria that may degrade numerous polysaccharides such as starch, cellulose, and xylan, and produce short-chain fatty acids (SCFAs). Moreover, they play a fundamental role in the biohydrogenation (BH) of dietary unsaturated fatty acids (UFAs) [42,43], but at the same time, they are susceptible to this type of fatty acids (FAs) [44]. A number of observations suggest that Ruminococcaceae and Christensenellaceae are potentially beneficial bacteria because they participate in the positive regulation of the intestinal environment and are linked to immunomodulation and healthy homeostasis [45]. 

In dairy goat, Fibrobacteriaceae has been correlated with FAs involved in the BH pathway of α-linolenic acid (C18:3n3-ALA) [35]. The increase in Fibrobacteriaceae in the OL+ group may be due to a higher intake of C18:3n3 which represents the major FA of OL [46]. In the rumen, different bacteria families are responsible for the BH of dietary UFAs. The BH process identified two important microbial transformations of fats in the rumen: lipolysis and BH. Bacteria belonging to the Acidaminococcaceae family, such as *Anaerovibrio lipolytica,* hydrolyze the ester bond of tri- and di-glycerides; on the contrary, *Butyrivibrio* (Lachnospiraceae family) lipases hydrolyze phospholipids. The *Butyrivibrio* group, including the genus *Butyrivibrio* and *Pseudobutyrivibrio*, and the species *B. proteoclasticus*, are able to hydrogenate C18:3 and form cis9, trans11-conjugated linoleic acid (C18:2, c9, t11-CLA) and vaccenic acid (C18:1, t11-VA) [47,48,49]. Previous studies carried out on different species of ruminants have reported that diets supplemented with agro-industrial by-products containing considerable amounts of plant secondary metabolites such as different kinds of polyphenols, saponins, and essential oils, are able to modulate rumen BH [50,51]. However, the effects on the rumen microbial population differ in relation to several factors such as the rumen passage rate, interaction with basal diet, different amounts of lipids in the diet, and the specific composition of phenolic substances. In this trial, no significant differences between the two groups were observed in Lachnospirace and Acidaminococcacea families, and the relative abundance of *Butyrivibrio, Pseudobutirivibrio*, and *Anaerovibrio* genera did not change, suggesting that OL dietary integration was not able to affect ruminal BH. However, further analysis is required to better understand the possible influence of OL intake on lipid metabolism. 

At the genera level, the results showed a higher abundance of *Paludibacter, Fibrobacter, Sphaerochaeta Christensenella, Rikenella, Oligosphaera, Candidatus Endomicrobium, Anaerovorax*, and *Atopobium* in the rumen of goat kids fed with OL; on the contrary, *Bacteroides* and *Selenomonas* were negatively influenced. In both groups, *Prevotella* (phylum Bacteroidetes) was the most dominant genus, in accordance with studies in goats and in other ruminants [52,53]. *Prevotella* comprises many species involved in the degradation of different substrates such as hemicelluloses, pectin, starch, protein, and simple sugars as energy sources to produce succinate as the major fermentation end product. In the present study, no significant differences between the two groups were observed in the abundance of *Prevotella*. These data were in accordance with previous studies where it has been demonstrated that the abundance of *Prevotella* did not always change in relation to different diets. Liu et al. [53] observed a higher abundance, although not significant, of *Prevotella* in goats fed a complete feed diet with respect to goats fed an all-forage diet. Bekele et al. [52] showed an increasing trend of *Prevotella* in the concentrate-fed group compared to the roughage group in the rumen of sheep, although the abundance did not differ significantly in the various ration groups. On the contrary, Huo et al. [54] found that the abundance of *Prevotella* in the rumen from hay-fed goats was higher than in concentrate-fed animals. 

Among the main fibrolytic bacteria (*Fibrobacter, Ruminoccoccus, Butyrivibrio*, and *Clostridium* spp.), significant variations were observed only in *Fibrobacter*, even if *Ruminococcus* spp. and *Clostridium* spp. were present at a higher relative abundance in the OL+ group with respect to the CTR. *Fibrobacter* is the major cellulose-degrading bacterial species in the rumen of herbivorous animals and is present in high-fiber diets [55]. Compared with other fibrolytic species such as *Ruminococcus*, it digests fiber faster. Different studies have demonstrated that in animals fed a tannin-rich diet, the fiber-degrading bacteria, *Fibrobacter* and *Ruminococcus*, were reduced [56]. In this study, a specific evaluation of tannins content in the used OL was not performed; however, this matrix was demonstrated to be relatively rich in these compounds [57]; therefore, it must be taken into account that the method of administration, the proportion of OL in the diet, and the time of the trial used in our study could have affected the observed effects on fibrolytic bacteria. 

Recently, it has been highlighted that bioactive plant compounds such as tannin and polyphenols can modify rumen methane production. Moreover, in vitro studies have shown a reduction in methane production in the rumen combined with different parts of olive [19]. Many methanogens belong to the phylum Euryarcheota (Archea domain) and the most common are from the genera *Methanobrevibacter*, *Methanimicrococcus*, and *Methanobacterium*, which convert H_2_ and CO_2_ into methane. Less abundant are *Methylotrophs*, *Methanosarcinales*, *Methanosphaera*, and *Methanomassiliicoccaceae* which produce methane using methylamines and methanol [11]. 

In our study, methanogens were scarcely represented, accounting for only 9.9 of Euryarcheota and 14.1 of the number of observed OTUs in CTR and OL+, respectively, with a relative abundance of about 0.01% in both groups. At deeper taxonomy levels, among methanogens, three different genera were detected in both groups: *Methanimicroccus* (Methanosarcinaceae family), *Methanobrevibacter* (Methanobacteriaces family), and *Methanomassiliicoccus* (Methanomassiloliicoccaceae family); however, their relative abundance was lower than 0.01%.

VOCs are produced by the metabolism of microorganisms that are able to ferment feed components. The diet can directly affect the rumen microbiota, and subsequently the production of VOCs. The specific composition of VOCs produced from a given bacterial community will depend on the diversity and metabolic types/groups of species present. The detection of these compounds in the rumen is a useful tool to evaluate physiological changes due to animal nutrition and numerous analytical approaches have been developed to accurately characterize and measure VOCs. 

In the present study, the headspace sampling with solid-phase microextraction coupled with gas chromatography-mass spectrometry was an effective approach that allowed to highlight 24 compounds belonging to different chemical families: acids, aldehydes, alcohols, ketones, esters, and hydrocarbons. First of all, the dietary supplementation seems to have induced an increase in hexanoic acid and a decrease in decanal at the rumen level. This condition assumes particular relevance when compared with what was previously reported by Spinhirne et al. [58], who exploited the solid-phase microextraction followed by gas chromatography-mass spectrometry for the analysis of volatile organic compounds in bovine breath. In particular, it was highlighted that decanal was more associated with clinically morbid steers, while the presence of hexanoic acid was symptomatic of healthy steers. As far as our study is concerned, it is therefore plausible that dietary supplementation with OL has somehow had positive effects on animal health. In support of what has been reported, the presence only in OL+ samples of α-terpineol, a monocyclic monoterpene tertiary alcohol naturally present in vegetable species, should also be highlighted. In fact, this compound is associated with a wide range of interesting biological properties for its antioxidant, anticancer, anticonvulsant, antiulcer, and antihypertensive potential; this compound was even credited with insecticidal properties [59]. 

Overall, what has been reported therefore lays the basis for hypothesizing an effect of dietary OL supplementation in improving the welfare conditions of the animals involved in the experimentation. In fact, the reduction in the straight-chain aldehydes derived from the oxidative degradation of UFAs is generally associated in mammals with a condition of reduced oxidative stress. Furthermore, the concomitance with the accumulation of hexanoic acid confirms this aspect as it indicates the predominance of triglycerides degradation following enzymatic events [60]. However, from this point of view, it is necessary to conduct further and more in-depth evaluations, which can clarify the metabolomic aspects associated with these findings, also in light of the changes observed at the metagenomic level.

## 5. Conclusions

Our data suggest the capability of OL to modulate the ruminal microbial community in goat kids. The OL addition seemed to improve fiber degradation modulating cellulolytic bacteria. The changes observed at the metagenomic level were associated with a different rumen volatile profile, and the presence of VOCs associated with health status suggests a favorable role of OL in preserving and improving animal welfare. Further investigations are needed to improve knowledge of the functional genomic framework of olive leaves degradation in the rumen and to examine their impact on lipid and biohydrogenation metabolism. 

## Figures and Tables

**Table 1:** Ingredient and chemical composition of the concentrate for control group (CTR) and experimental group (OL+).

	CTR	OL+
*Ingredients (%)*		
Soybean, meal	17.50	15.50
Wheat, bran	20.00	20.00
Barley, meal	32.00	27.00
Corn, meal	28.50	25.50
Olive leaves	-	10.00
Vitamin and mineral	2.00	2.00
*Chemical composition (%)*		
Dry matter (DM)	89.10	88.50
Ash ^1^, %	5.10	5.26
Ether extract (EE) ^1^, %	3.25	3.36
Crude protein (CP) ^1^, %	18.20	17.95
Neutral detergent fiber (NDF) ^1^, %	12.55	13.32
Acid detergent fiber (ADF) ^1^, %	5.44	5.95
Acid detergent lignin (ADL) ^1^, %	1.16	1.39
Starch ^1^, %	48.59	43.88
ME ^1^, MJ/kg	7.47	7.18

^1^ on a DM basis.

**Table 2:** Data acquisition for control group (CTR) and experimental group (OL+).

	CTR	OL+
Starting reads	236,831 ± 114,396	277,576 ± 48,923
Trimmed	220,392 ± 110,678	257,589 ± 47,028
Filtered	219,302 ± 110,140	256,299 ± 46,772
Denoised R1	218,575 ± 109,722	255,226 ± 46,491
Denoised R2	218,290 ± 109,463	254,648 ± 46,359
Merged	217,631 ± 109,077	253,679 ± 46,116
Non-chimera	1,955,481 ± 91,487	221,149 ± 42,834
Reads for analysis (%)	83	80

Data are reported as the mean of the ten records per group ± standard deviation.

**Table 3:** SIMPER-generated genus list ordered by percentage contribution to Bray-Curtis dissimilarity and genera of which abundances significantly differ (OL+ vs CTR) based on Kruskal–Wallis tests.

Genus	Average	S.D.	Ratio	CTR	OL+	Cumulative Sum	*p*-Value	*q*-Value (≤0.05)
*Prevotella*	0.0691659	0.0399527	1.7312	9207.3	8482.8	0.1828	0.290	
*Subdivision5_genera_incertae_sedis*	0.0490198	0.0492473	0.9954	1759	3719.2	0.3124	0.023	
*Paraprevotella*	0.040015	0.0494914	0.8085	2459.2	790.5	0.4182	0.019	
*Oscillibacter*	0.0256097	0.0256221	0.9995	937.6	1385.1	0.4859	0.070	
*Ruminobacter*	0.0248105	0.0331442	0.7486	1207.4	326	0.5515	0.050	
*Anaerocella*	0.022674	0.0263719	0.8598	1033.3	33	0.6115	0.041	
***Paludibacter***	**0.0202838**	**0.0106447**	**1.9055**	**259**	**1061.5**	**0.6651**	**0.003**	**0.027**
***Fibrobacter***	**0.0166172**	**0.0120337**	**1.3809**	**849.2**	**1502.7**	**0.709**	**0.010**	**0.044**
*Anaerobacterium*	0.0131868	0.0117324	1.124	728	204.9	0.7439	0.082	
*Ruminococcus*	0.0122287	0.0090614	1.3495	584.9	991.6	0.7762	0.034	
*Anaeroplasma*	0.0096275	0.0118004	0.8159	453.2	270.3	0.8016	0.384	
***Sphaerochaeta***	**0.0068604**	**0.0046886**	**1.4632**	**142.3**	**386.2**	**0.8198**	**0.004**	**0.026**
*Treponema*	0.0067165	0.0040503	1.6583	574.2	623.6	0.8375	0.705	
*Butyrivibrio*	0.0059045	0.0060017	0.9838	373.7	266.2	0.8531	0.677	
*Acetobacteroides*	0.0053678	0.0087699	0.6121	38.4	224.9	0.8673	0.249	
*Saccharibacteria_genera_incertae_sedis*	0.0048374	0.0052325	0.9245	260.9	132.2	0.8801	0.406	
*Clostridium IV*	0.0044499	0.0049431	0.9002	129	268.9	0.8919	0.112	
*Lachnospiracea_incertae_sedis*	0.003994	0.0029685	1.3454	313.7	298	0.9024	0.520	
***Bacteroides***	**0.0039234**	**0.0023095**	**1.6988**	**233.2**	**67.3**	**0.9128**	**0.003**	**0.023**
*Succiniclasticum*	0.0036021	0.0045189	0.7971	174.2	56.6	0.9223	0.173	
***Christensenella***	**0.0029613**	**0.0020312**	**1.4579**	**76.1**	**192.9**	**0.9302**	**0.013**	**0.049**
*Mogibacterium*	0.0029331	0.0024578	1.1934	106.9	170.3	0.9379	0.151	
***Rikenella***	**0.0029132**	**0.0018389**	**1.5842**	**44.4**	**165.7**	**0.9456**	**0.005**	**0.032**
*Clostridium XlVa*	0.0022393	0.0020527	1.0909	29.3	118.9	0.9515	0.07	
*Saccharofermentans*	0.0021554	0.0012938	1.6659	121.8	168.3	0.9572	0.034	
*Pseudobutyrivibrio*	0.0020747	0.0014231	1.4579	128.9	60.7	0.9627	0.082	
***Oligosphaera***	**0.0019913**	**0.0015864**	**1.2552**	**49.7**	**137**	**0.968**	**0.008**	**0.040**
*Succinivibrio*	0.0018624	0.001527	1.2196	83.4	69.8	0.9729	0.733	
***Candidatus Endomicrobium***	**0.0016373**	**0.0013349**	**1.2266**	**3.4**	**77.6**	**0.9772**	**0.0001**	**0.005**
***Anaerovorax***	**0.0013937**	**0.0010073**	**1.3836**	**12.3**	**71.3**	**0.9809**	**0.002**	**0.036**
*Olsenella*	0.0011188	0.0006048	1.8497	44.4	75.4	0.9839	0.29	
*Flavonifractor*	0.0010989	0.0014461	0.7599	49.7	0.8	0.9868	0.49	
*Victivallis*	0.0010159	0.001484	0.6846	19.2	58.8	0.9895	0.069	
*Vampirovibrio*	0.0008977	0.0006995	1.2833	46.5	29.8	0.9918	0.650	
***Selenomonas***	**0.0007026**	**0.0004087**	**1.7191**	**43**	**11.6**	**0.9937**	**0.003**	**0.036**
***Atopobium***	**0.0006991**	**0.0005682**	**1.2303**	**12.7**	**40.6**	**0.9956**	**0.014**	**0.049**
*Elusimicrobium*	0.000696	0.0004842	1.4373	33.8	51.5	0.9974	0.211	
*Blautia*	0.0005036	0.0003512	1.434	21.6	33.7	0.9987	0.211	
*Intestinimonas*	0.0004819	0.0004932	0.9772	14.2	32.8	1	0.017	

In bold: genus for which the abundance significantly differed (*p* < 0.05) between the two groups based on Kruskal–Wallis non-parametric ANOVA tests.

**Table 4:** SIMPER-generated family list ordered by percentage contribution to Bray-Curtis dissimilarity and families of which abundances significantly differ between two groups (OL+ vs CTR) based on Kruskal–Wallis tests.

Families	Average	S.D.	Ratio	CTR	OL+	Cumulative Sum	*p*-Value	*q*-Value (≤ 0.05)
Prevotellaceae	0.058200	0.035000	1.6624	10449	8134.1	0.2359	0.096	
Subdivision5_genera_incertae_sedis	0.031800	0.031900	0.9972	1563.9	3041.7	0.3648	0.023	
**Succinivibrionaceae**	**0.026700**	**0.028900**	**0.9233**	**1699.2**	**347.1**	**0.4729**	**0.005**	**0.028**
Ruminococcaceae	0.024600	0.017300	1.4234	4425.6	5237.3	0.5724	0.290	
Porphyromonadaceae	0.024500	0.021900	1.118	3670.3	4806.1	0.6716	0.112	
Rikenellaceae	0.017900	0.017000	1.057	1068.7	268.6	0.7443	0.140	
Lachnospiraceae	0.015400	0.010000	1.5428	1983	2245.7	0.8069	0.650	
**Fibrobacteraceae**	**0.011200**	**0.008560**	**1.3092**	**684.2**	**1249.5**	**0.8523**	**0.010**	**0.041**
Spirochaetaceae	0.007300	0.005480	1.3321	754.7	934.7	0.8819	0.290	
Anaeroplasmataceae	0.006220	0.007280	0.8532	383.9	248.8	0.9071	0.910	
Acidaminococcaceae	0.003170	0.005220	0.6065	191.6	50.5	0.9199	0.174	
Clostridiales_Incertae Sedis XIII	0.003160	0.002490	1.2672	115.1	223.9	0.9328	0.034	
Saccharibacteria_genera_incertae_sedis	0.003000	0.002850	1.0543	195.8	109.4	0.9449	0.571	
Bacteroidaceae	0.002530	0.001650	1.5293	185.7	72.6	0.9552	0.015	
**Christensenellaceae**	**0.002150**	**0.001450**	**1.4783**	**71.7**	**165.2**	**0.9639**	**0.006**	**0.030**
**Coriobacteriaceae**	**0.001590**	**0.000809**	**1.9603**	**67.3**	**131.5**	**0.9703**	**0.003**	**0.018**
**Oligosphaeraceae**	**0.001550**	**0.001070**	**1.4428**	**39**	**121.3**	**0.9766**	**0.002**	**0.015**
**Candidatus Endomicrobium**	**0.001050**	**0.000740**	**1.425**	**2.6**	**61.1**	**0.9808**	**0.0001**	**0.004**
Erysipelotrichaceae	0.000655	0.000820	0.7987	25.5	54.2	0.9835	0.363	
**Planctomycetaceae**	**0.000635**	**0.000791**	**0.8022**	**0.9**	**36.1**	**0.9861**	**0.001**	**0.008**
Victivallaceae	0.000624	0.000857	0.7283	18.9	46.4	0.9886	0.272	
Bdellovibrionaceae	0.000541	0.000460	1.1771	28.8	25.7	0.9908	0.597	
Elusimicrobiaceae	0.000535	0.000386	1.3869	31.9	44.5	0.993	0.427	
Veillonellaceae	0.000431	0.000313	1.379	50.5	32.2	0.9947	0.064	
Verrucomicrobiaceae	0.000252	0.000760	0.3317	0	14	0.9957	0.317	
Eubacteriaceae	0.000240	0.000217	1.1074	10.6	20.8	0.9967	0.120	
Rhodospirillaceae	0.000170	0.000255	0.6662	9.3	5.5	0.9974	0.85	
Acholeplasmataceae	0.000152	0.000160	0.952	7.6	3.8	0.998	0.831	
Sutterellaceae	0.000151	0.000169	0.8924	2.1	9.6	0.9986	0.029	
**Bifidobacteriaceae**	**0.000132**	**0.000108**	**1.2206**	**7.3**	**0**	**0.9992**	**0.0002**	**0.003**
Streptococcaceae	0.000126	0.000089	1.4141	7.3	6.4	0.9997	0.210	
Anaerolineaceae	0.000083	0.000073	1.1437	3	6.7	1	0.207	

In bold: families for which the abundance significantly differed (*p* < 0.05) between the two feed groups based on Kruskal–Wallis non-parametric ANOVA tests.

**Table 5:** Volatile compounds (VOCs) detected in rumen samples obtained from goat kids fed a standard diet (CTR) and animals fed a dietary supplementation with olive leaves (OL+).

VOCs ^1^	CTR	OL+	*p*-Value
**Acids**			
Acetic acid	3.58 ± 0.49	3.12 ± 0.45	ns
Butanoic acid	40.83 ± 3.94	36.03 ± 3.79	ns
Butanoic acid, 3-methyl	4.02 ± 0.32	4.24 ± 0.35	ns
Pentanoic acid	0.81 ± 0.09	0.91 ± 0.11	ns
Hexanoic acid	7.07 ± 0.76	9.35 ± 0.89	*
Eicosanoic acid	0.41 ± 0.05	0.40 ± 0.06	ns
**Aldehydes**			
Pentanal, 2-methyl	6.17 ± 0.57	6.21 ± 0.76	ns
Decanal	1.89 ± 0.17	0.85 ± 0.10	**
Undecanal	0.39 ± 0.05	0.33 ± 0.04	ns
Tridecanal	0.24 ± 0.04	0.22 ± 0.03	ns
Hexadecanal	1.44 ± 0.13	1.33 ± 0.16	ns
Pentadecanal	1.08 ± 0.14	1.00 ± 0.12	ns
**Alcohol**			
2-hexanol	1.00 ± 0.09	0.88 ± 0.09	ns
α-terpineol	nd	3.06 ± 0.26	**
**Ketones**			
2-nonanone	5.47 ± 0.47	5.57 ± 0.53	ns
2-hexanone, 5-methyl	0.26 ± 0.03	0.27 ± 0.03	ns
2-heptadecanone	0.23 ± 0.02	0.31 ± 0.04	ns
**Esters**			
Pentanoic acid, ethyl ester	8.82 ± 0.95	10.31 ± 0.93	ns
Heptanoic acid, ethyl ester	1.27 ± 0.11	1.11 ± 0.10	ns
Octadecanoic acid, phenylmethyl ester	0.80 ± 0.08	0.71 ± 0.08	ns
Eicosanoic acid, phenylmethyl ester	0.46 ± 0.05	0.48 ± 0.04	ns
Oxalic acid, allyl octadecyl ester	0.21 ± 0.03	0.19 ± 0.02	ns
**Hydrocarbons**			
Octadecane, 3-ethyl-5-(2-ethylbutyl)	2.06 ± 0.23	1.82 ± 0.17	ns
Octanoic acid	1.04 ± 0.13	1.09 ± 0.08	ns

^1^ Volatile compounds (VOCs) are expressed as relative mean percentages (%) of total detected compounds ± standard deviation (SD). nd = not detectable; ns = not significant; * *p* < 0.05; ** *p* < 0.01.

## Data Availability

All data are available following reasonable request to the corresponding author.

## Supplementary Materials

The following supporting information can be downloaded at: https://www.mdpi.com/article/10.3390/vetsci9090452/s1, File S1: Beta diversity; Table S1: Relative abundance (%) of the main genera identified in the control group (CTR) and experimental group (OL+).
