# Supplementary material for: Metagenomic Characterization and Volatile Compounds Determination in Rumen from Saanen Goat Kids Fed Olive Leaves
Source: Vet Sci. 2022 Aug 24;9(9):452. doi: 10.3390/vetsci9090452 (PMC9505022; doi:10.3390/vetsci9090452)
Supplement: Supplementary file 1 [file vetsci-09-00452-s001.zip › Table S1.pdf]

Table S1. Relative abundance (%) of the main genera identified in the control group (CTR) and experimental group (OL+).

**Genera**	**CTR**	**OL+**	P-value
Prevotella	40.089	36.913	0.5457
Subdivision5_genera_incertae_sedis	7.684	16.185	0.0282
Paraprevotella	10.802	3.416	0.0454
Fibrobacter	3.668	6.526	0.0064
Oscillibacter	4.091	6.043	0.4069
Ruminococcus	2.542	4.303	0.0400
Ruminobacter	5.281	1.431	0.1238
Paludibacter	1.115	4.655	0.0016
Treponema	2.535	2.736	0.6969
Anaerocella	4.544	0.146	0.0229
Anaerobacterium	3.169	0.933	0.0216
Anaeroplasma	2.012	1.203	0.4247
Butyrivibrio	1.612	1.139	0.3840
Lachnospiracea_incertae_sedis	1.358	1.265	0.7788
Sphaerochaeta	0.612	1.699	0.0173
Clostridium IV	0.568	1.139	0.1560
Saccharibacteria_genera_incertae_sedis	1.144	0.561	0.1961
Bacteroides	1.017	0.303	0.0012
Saccharofermentans	0.525	0.772	0.1204
Mogibacterium	0.473	0.724	0.3026
Christensenella	0.349	0.836	0.0119
Acetobacteroides	0.154	0.968	0.1930
Succiniclasticum	0.757	0.234	0.1271
Rikenella	0.187	0.710	0.0017
Oligosphaera	0.214	0.617	0.0011
Pseudobutyrivibrio	0.557	0.257	0.0291
Succinivibrio	0.367	0.316	0.7520
Clostridium XlVa	0.125	0.535	0.0149
Olsenella	0.194	0.324	0.0784
Elusimicrobium	0.167	0.210	0.3628
Anaerovorax	0.052	0.309	0.0023
Candidatus Endomicrobium	0.013	0.346	0.0010
Victivallis	0.089	0.246	0.1161
Vampirovibrio	0.191	0.136	0.4328
Selenomonas	0.189	0.053	0.0001
Blautia	0.088	0.147	0.1075
Atopobium	0.058	0.173	0.0157
Flavonifractor	0.220	0.004	0.0403
Intestinimonas	0.061	0.146	0.0366
Stomatobaculum	0.009	0.174	0.0021
Alloprevotella	0.073	0.109	0.2044
Eubacterium	0.058	0.118	0.0643

Coprococcus	0.020	0.155	0.0000
Barnesiella	0.109	0.016	0.2297
Asteroleplasma	0.118	0.001	0.1450
Succinimonas	0.119	0.000	0.1361
Syntrophococcus	0.046	0.066	0.4857
Anaerorhabdus	0.005	0.091	0.0054
Roseburia	0.040	0.053	0.7145
Sporobacter	0.017	0.054	0.3102
Streptococcus	0.040	0.030	0.4916
Clostridium XlVb	0.042	0.027	0.3050
Bulleidia	0.007	0.057	0.0003
Acholeplasma	0.032	0.021	0.5312
Slackia	0.016	0.029	0.1252
Bifidobacterium	0.044	0.000	0.0006
Lachnobacterium	0.039	0.000	0.0378
Moryella	0.018	0.021	0.8041
Ethanoligenens	0.012	0.026	0.1882
Anaerovibrio	0.025	0.010	0.0042
Schwartzia	0.005	0.029	0.0858
Phocaeicola	0.013	0.021	0.7102
Aestuariispira	0.019	0.014	0.6658
Kandleria	0.031	0.000	0.0100
Acetatifactor	0.010	0.021	0.5454
Sutterella	0.008	0.017	0.4340
Victivallis	0.022	0.003	0.1089
Methanobrevibacter	0.012	0.012	0.9934
Lactobacillus	0.024	0.000	0.0854
Papillibacter	0.003	0.018	0.0068
Streptophyta	0.008	0.011	0.3460
Desulfuromonas	0.000	0.017	0.0106
Pseudoflavonifractor	0.000	0.013	0.2772
Campylobacter	0.008	0.005	0.3089
Snodgrassella	0.007	0.005	0.5179
Howardella	0.006	0.004	0.5870
Pseudoscardovia	0.010	0.000	0.1470
Parasutterella	0.001	0.008	0.0668
Methylobacterium	0.007	0.002	0.2219
Acetanaerobacterium	0.002	0.006	0.4352
Lactonifactor	0.000	0.008	0.1369
Enterorhabdus	0.003	0.005	0.4646
SR1_genera_incertae_sedis	0.003	0.004	0.2595
Anaerofustis	0.001	0.006	0.0076
Methanomassiliicoccus	0.002	0.004	0.0382
Denitrobacterium	0.001	0.005	0.0043
Phascolarctobacterium	0.000	0.004	0.3306

Blastopirellula	0.001	0.003	0.2184
Desulfovibrio	0.002	0.001	0.5540
Hydrogenoanaerobacterium	0.001	0.002	0.1616
Pyramidobacter	0.003	0.000	0.0600
Coriobacterium	0.001	0.001	0.9185
Acinetobacter	0.001	0.002	0.2138
Dorea	0.000	0.002	0.3306
Defluviitalea	0.000	0.002	0.0014
Actinomyces	0.001	0.001	0.3518
Oribacterium	0.002	0.000	0.1408
Methanimicrococcus	0.000	0.002	0.0081
Ruminococcus2	0.002	0.000	0.2539
Lautropia	0.000	0.002	0.0015
Catabacter	0.000	0.002	0.0676
Neisseria	0.001	0.001	0.8229
Faecalibacterium	0.002	0.000	0.3306
Chryseobacterium	0.002	0.000	0.4375
Moraxella	0.002	0.000	0.3942
Desulfobulbus	0.001	0.001	0.7291
Brachymonas	0.001	0.000	0.0630
Eisenbergiella	0.001	0.001	0.8732
Centipeda	0.000	0.002	0.3306
Lachnoanaerobaculum	0.001	0.001	0.5705
Anaerotruncus	0.000	0.001	0.1818
Anaerobiospirillum	0.001	0.001	0.9665
Fretibacterium	0.001	0.001	0.6802
Pseudomonas	0.001	0.001	0.8651
Alysiella	0.000	0.001	0.0681
Corynebacterium	0.001	0.000	0.3066
Staphylococcus	0.000	0.001	0.6150
Pasteurella	0.001	0.001	0.9688
Others	0.004	0.004	0.086
